# Dietary Geraniol by Oral or Enema Administration Strongly Reduces Dysbiosis and Systemic Inflammation in Dextran Sulfate Sodium-Treated Mice

**DOI:** 10.3389/fphar.2016.00038

**Published:** 2016-03-03

**Authors:** Luigia De Fazio, Enzo Spisni, Elena Cavazza, Antonio Strillacci, Marco Candela, Manuela Centanni, Chiara Ricci, Fernando Rizzello, Massimo Campieri, Maria C. Valerii

**Affiliations:** ^1^Biology Unit, Department of Biological, Geological and Environmental Sciences, University of BolognaBologna, Italy; ^2^Department of Pharmacy and Biotechnology, University of BolognaBologna, Italy; ^3^Department of Clinical and Experimental Sciences, University of BresciaBrescia, Italy; ^4^Department of Medical and Surgical Sciences, University of BolognaBologna, Italy

**Keywords:** geraniol, inflammatory bowel disease (IBD), dextran sulfate sodium (DSS)-induced colitis, cyclooxygenase-2 (COX-2), inflammation, dysbiosis

## Abstract

(Trans)-3,7-Dimethyl-2,6-octadien-1-ol, commonly called geraniol (Ge-OH), is an acyclic monoterpene alcohol with well-known anti-inflammatory, antitumoral, and antimicrobial properties. It is widely used as a preservative in the food industry and as an antimicrobial agent in animal farming. The present study investigated the role of Ge-OH as an anti-inflammatory and anti-dysbiotic agent in the dextran sulfate sodium (DSS)-induced colitis mouse model. Ge-OH was orally administered to C57BL/6 mice at daily doses of 30 and 120 mg kg^(−1)^ body weight, starting 6 days before DSS treatment and ending the day after DSS removal. Furthermore, Ge-OH 120 mg kg^(−1)^ dose body weight was administered via enema during the acute phase of colitis to facilitate its on-site action. The results show that orally or enema-administered Ge-OH is a powerful antimicrobial agent able to prevent colitis-associated dysbiosis and decrease the inflammatory systemic profile of colitic mice. As a whole, Ge-OH strongly improved the clinical signs of colitis and significantly reduced cyclooxygenase-2 (COX-2) expression in colonocytes and in the gut wall. Ge-OH could be a powerful drug for the treatment of intestinal inflammation and dysbiosis.

## Introduction

More than 90% of the 100 trillion cells in the human body are microbes, most of which reside in the digestive tract and are collectively known as the intestinal microbiota (Yaung et al., 2014). The bacterial flora is extremely dense and diverse and shapes fundamental physiological processes such as digestion and the development of gut-associated lymphoid tissues and systemic immunity. The intestinal microbiota plays a crucial role in maintaining colonic homeostasis, while microbial dysbiosis can contribute to a wide spectrum of disease (Kamada et al., 2013).

Inflammatory bowel disease (IBD), which includes Crohn's disease (CD), and ulcerative colitis (UC), is a chronic inflammatory disorder of the intestinal tract associated with abdominal pain, intestinal bleeding, weight loss, and diarrhea (Koloski et al., 2008). The etiology of IBD is unknown but the one dominant hypothesis is that the inflammation results from altered or pathogenic microbiota in a genetically susceptible host. A growing body of literature implicates the abnormal overgrowth or dominance of particular bacterial species in the pathogenesis of IBD. Notably, mouse model studies of IBD have shown protection against the development of IBD in a germ-free environment, corroborating the role of gut flora in the pathogenesis of this spectrum of illnesses (Missaghi et al., 2014).

As in humans, the two most abundant bacterial phyla in C57BL6/J mice are the *Firmicutes* (60–80% of sequences) and the *Bacteroidetes* (20–40%). *F*ew bacteria are present in the mouse gut soon after birth. The neonate is inoculated with microorganisms by the mother and the environment and the microbiota is fully established when the mouse reaches adulthood at around 8 weeks, even if it is still susceptible to changes in composition (Laukens et al., 2015). In healthy adults, diet changes remain the major player in microbiota dynamics.

Essential oil mixtures have been shown to play a significant role in the modulation of animal gut microbiota (Oviedo-Rondón et al., 2006) but their mechanism(s) of action remain incompletely understood (Thompson et al., 2013).

Essential oils (EO) are volatile natural complex compounds characterized by a strong odor and synthesized by aromatic plants as secondary metabolites (Bakkali et al., 2008). They are highly complex natural mixtures which may contain up to 60 components at widely varying concentrations. In nature, EO play important roles in the protection of plants acting as antibacterial, antiviral, antifungal, and insecticidal agents (Bakkali et al., 2008; Fang et al., 2010). Recently, EO have been used in animal feed to treat infections, manipulate gut fermentation, and improve productivity (Wallace et al., 2010).

Geraniol (Ge-OH) is a naturally acyclic monoterpene component of EO extracted from lemongrass, rose, and other aromatic plants. Several studies on the biological activities of Ge-OH have shown it to be a highly active antitumoral, antimicrobial compound, with antioxidant and anti-inflammatory properties (Ahmad et al., 2011; Thapa et al., 2012; Khan et al., 2013).

Ge-OH's antimicrobial activities do not seem to have specific cellular targets. Like other EO, Ge-OH is a hydrophobic compound able to bind to the bacterial wall modifying its dynamic organization, with a consequent loss of ions and ATP depletion (Di Pasqua et al., 2006; Turina et al., 2006). In addition to bacterial growth inhibition, especially effective on Gram-positive bacteria (Thapa et al., 2012), Ge-OH also damages bacterial proteins, and lipids (Burt, 2004; Oussalah et al., 2007). Ge-OH effectively modulates the drug resistance of several Gram-negative bacterial species such as *E. aerogenes, E. coli*, and *P. aeruginosa* by restoring drug susceptibility in strains overexpressing efflux pumps (Solórzano-Santos and Miranda-Novales, 2012). It is important to emphasize that human pathogenic bacteria are more sensitive to Ge-OH than are commensal species even if the nature of this selectivity remains unsettled (Singh et al., 2012).

Ge-OH has antioxidant activities in eukaryotic cells (Khan et al., 2013). By reducing oxidative stress, Ge-OH may prevent drug-induced mitochondrial dysfunction in hepatocytes (Singh et al., 2012). *In vivo*, it proved able to enhance neurodegeneration in a mice model of Parkinson's disease (Rekha et al., 2013).

*In vitro* and *in vivo*, Ge-OH inhibits the expression of cyclooxygenase-2 (COX-2; Chaudhary et al., 2013), a key enzyme in inflammation (Strillacci et al., 2010). The anti-inflammatory properties of Ge-OH have been assessed on different animal models and in this context it has been shown that its molecular target is not only COX-2 but also NF-kB (Marcuzzi et al., 2011; Khan et al., 2013; Medicherla et al., 2015).

Considering all its activities, Ge-OH seems to be an excellent candidate for the treatment of gut and systemic inflammations and for the control of gut dysbiosis. Medicherla et al. (2015) have already proved that Ge-OH effectively modulates experimental colitis, but the possibility of using this molecule as a therapeutic agent has yet to be demonstrated. Their study did not consider the chemical characteristics of Ge-OH that require specific formulations to be administered. They administered Ge-OH orally diluted in saline, forgetting that Ge-OH is insoluble in aqueous solutions in which it rapidly tends to separate from the water. Moreover, once separated from water, Ge-OH reaches high concentrations at which it could irritate the gut mucosa. Since, this substance rapidly crosses enterocyte monolayers (Heinlein et al., 2014), its site of action and its impact on the microbiota should also be evaluated. To determine whether Ge-OH could become a therapeutic option in humans, we administered Ge-OH in appropriate oral or enema formulations to dysbiotic mice and compared its effects with one of the standard therapies currently used to manage gut inflammation in IBD patients.

## Materials and methods

### Ge-OH oral formulation

Ge-OH oral formulation was optimized for the administration route chosen and for a possible transition to use in humans as it has a strong smell and very unpleasant taste. In addition, Ge-OH is completely water insoluble and could irritate the mucosae if administered pure. The oral formulation was then optimized for a slow release of Ge-OH using a patented soy lecithin incapsulation. Natural Ge-OH (analytical grade, >98% pure) and soy lecithin were purchased from Prodasynth (Grasse, France). All the other reagents were purchased from SIGMA-Aldrich (St Louis, MO, USA). The stable suspension was prepared by Cedax Srl (Forlì, Italy) by adding Ge-OH (ρ = 0.899 g/cm^3^; 17% by weight) to a solution containing sucrose (16%), deionized water (22%) and soy lecithin (25%), and ethanol (20%) as preservative (patent PCT WO 201 1/128597). The suspension was stored at 4°C and administered by oral gavage to Ge-OH-treated mice (4, 5, or 18 μl of Ge-OH suspension brought to the final volume of 100 μl with Ge-OH-free suspension). A Ge-OH-free suspension containing sucrose (16%), soya lecithin (25%), and ethanol (20%) was administered to the control group.

### Ge-OH enema formulation

The Ge-OH formulation for enema administration was prepared using glycerin to increase the viscosity of the solution and thereby facilitate both intracolonic injection and colonic retention. Enema Ge-OH solution was prepared as follows: natural Ge-OH (4% v/v) was added to a solution containing PBS and glycerol (30%v/v). An amount of solution corresponding to 120 mg kg^(−1)^ (body weight, die) was freshly prepared and administered by enema during the acute phase of colitis. A control solution mixed as previously described but without Ge-OH was also prepared and administered to the enema control group. Enema treatments were administered via a 16G venous catheter (diameter 2 mm, length 48 mm; BD Bioscience, Buccinasco, Italy) advanced through the rectum into the colon until the tip was 10 mm proximal to the anus. A venous catheter was applied to a 1 ml syringe and the suspension was gently injected into the rectum. Animals were sedated using tiletamine 10 mg kg^(−1)^ plus xylazine 2.5 mg kg^(−1)^ during enema administration.

### Hydrocortisone enema treatment

Enema administrations were prepared as follows: hydrocortisone (0.08% w/v, Sigma) was added to a solution containing PBS and glycerol (30%v/v). An amount of solution corresponding to 2.5 mg kg^(−1)^ (body weight, die) was freshly prepared and administered by enema during the acute phase of colitis. Enema treatments were administered via a 16G venous catheter as previously described.

### Animal treatment

Sixty-four eight-week-old male C57BL/6 mice were purchased from Charles River Laboratories (Lecco, Italy). Animals were housed in collective cages with a controlled environment containing two mice each, at 22 ± 2°C and 50% humidity, under a 12-h light/dark cycle. Mice were allowed to acclimate to these conditions for at least 7 days before inclusion in experiments and had free access to food and water throughout the study.

Mice were randomized into eight experimental groups: the first (I) group called CTRL (*n* = 8) received only tap water for 37 days (1–37). Group II called SoySusp-or received only tap water for 37 days (1–37) and mice were treated with oral Ge-OH-free suspension for 17 days (days 8–24) to analyze possible modifications of the microbiota induced by the soy lecithin suspension in healthy mice. All the groups III–VIII received tap water for 16 days (1–16), oral administration of 1.5% (w/v) dextran sulfate sodium (DSS for colitis, TdB Consultancy, Sweden) for 7 days in tap water (days 17–23), and tap water for 14 days (25–37). DSS was freshly prepared every 7 days and the average amount of DSS taken was recorded daily. In addition to DSS, group III, called DSS (*n* = 8), received the control oral Ge-OH-free suspension for 17 days (days 8–24), while group IV, called DSS+Ger30 or (*n* = 8), received Ge-OH orally [30 mg kg^(−1)^] for 17 days (days 8–24). Group V, called DSS+Ger120 or (*n* = 8), received Ge-OH [120 mg kg^(−1)^] for 17 days (days 8–24), and group VI (*n* = 8), called DSS+Ger120 En, received four enema administrations of Ge-OH on experimental days 19, 21, 23, and 25. Group VII (*n* = 8), called DSS+Susp enema, received four enema administrations of glycerol-PBS suspension on days 19, 21, 23, and 25.

The last group, VIII, called DSS+hydrocortisone enema, received four enema administrations of glycerol-PBS-hydrocortisone on days 19, 21, 23, and 25. This group was used as a model to understand how colitis is clinically modulated by a powerful drug. Hydrocortisone was administered by enema at doses of 2.5 mg kg^(−1)^ body weight.

**Figure 1:**
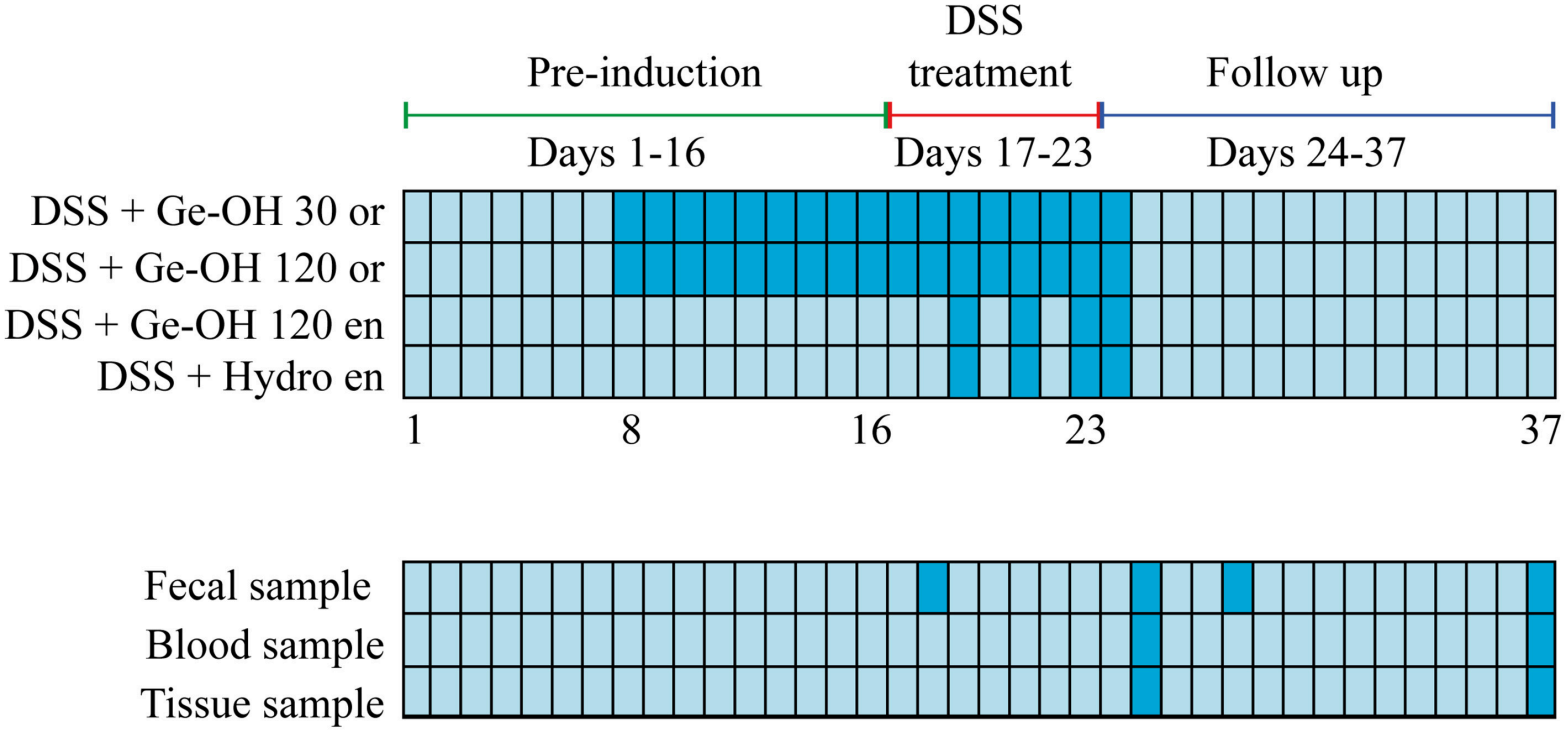
**Experimental design of the study**. Animal treatment and the collection of feces, blood, and tissue are indicated (dark blue) in the grid.

The experimental design is schematized in Figure 1. The experiments were carried out in accordance European and Italian guidelines. They were approved by the Institutional Ethical Review Board of the University of Bologna and by the Italian Ministry for Research and were repeated twice.

### Disease activity index (DAI)

DAI was calculated by the combined score of weight loss, stool consistency and bleeding, as detailed in Table 1. All parameters were scored from day 1 to day 37.

**Table 1:** **Disease activity index (DAI) score parameters**.

**Stool consistency**	**Bleeding**	**Weight loss**
0 = Formed	0 = Normal color stool	0 = No weight loss
1 = Mild-soft	1 = Brown color	1 = 5–10% weight loss
2 = Very soft	2 = Reddish color	2 = 11–15% weight loss
3 = Watery stool	3 = Bloody stool	3 = 16–20% weight loss
		4 = >20% weight loss

### Histological evaluation of colitis

Mice (*n* = 2 for each experimental group) were anesthetized using Zoletil-100 [10 mg kg^(−1)^; Virbac, Carros, France], and Xilor [2.5 mg kg^(−1)^; Bio98, Milan, Italy] by intramuscular injection and sacrificed by cervical dislocation on day 25 (2 days after the end of DSS treatment, when the maximum DAI score was reached), and day 37, at the end of weight recovery. The colon was excised, rinsed with saline solution, fixed in 4% formalin and embedded in paraffin. Four micrometer sections were stained with hematoxylin-eosin and observed for histological assessment of epithelial damage by a pathologist in a blinded manner.

### Determination of plasma cytokine levels

Blood samples (200 μl) were taken from the tail vein on days 25 and 37 and collected in Eppendorf tubes. Blood was centrifuged at 1000 rpm for 10 min, and plasma was collected and stored at −80°C until BioPlex analysis. Cytokine levels were determined using a multiplexed mouse bead immunoassay kit (Bio-Rad, CA, USA). The six-plex assays (IL-1β, IL-6, IL-10, IL-17A, IFNγ, TNFα) were performed in 96-well plates following the manufacturer's instructions. Microsphere magnetic beads coated with monoclonal antibodies against the different target analytes were added to the wells. After 30 min incubation, the wells were washed and biotinylated secondary antibodies were added. After incubation for 30 min, beads were washed and then incubated for 10 min with streptavidin-PE conjugated to the fluorescent protein, phycoerythrin (streptavidin/phycoerythrin). After washing, the beads (a minimum of 100 per analyte) were analyzed in the BioPlex 200 instrument (BioRad). Sample concentrations were estimated from the standard curve using a fifth-order polynomial equation and expressed as pg/ml after adjusting for the dilution factor (Bio-Plex Manager software 5.0). The sensitivities of the assay were 3.14 pg/ml (IL-1β), 1.34 pg/ml (IL-6), 1.38 pg/ml (IL-10), 2.38 pg/ml (IL-17), 1.38 pg/ml (IFNγ), and 2.73 pg/ml (TNFα). Samples below the detection limit of the assay were recorded as zero. The intra-assay CV was <14%.

### Characterization of the intestinal microbiota by HTF-microbi.array

The intestinal mice microbiota was characterized using the fully validated diphylogenetic DNA microarray platform HTF-Microbi.Array. Targeting 33 phylogenetically related groups, this LDR-based universal array covers up to 95% of the mammalian gut microbiota. Gut microbiota analysis was performed on days 18, 25, 29, and 38. Total DNA from fecal material was extracted using the QIAamp DNA Stool Mini Kit (Qiagen) according to the modified protocol previously reported (Candela et al., 2010, 2012). Final DNA concentration was determined using NanoDrop ND-1000 (NanoDrop Technologies). A nearly full-length portion of 16S rDNA gene was amplified using universal forward primer 27F and reverse primer 1492R, according to the protocol previously described (Castiglioni et al., 2004) PCR amplifications were performed in a Biometra Thermal Cycler T Gradient (Biometra, Göttingen, Germany). PCR products were purified using the High Pure PCR Cleanup Microkit (Roche, Mannheim, Germany), eluted in 30 μl of sterile water and quantified with NanoDrop ND-1000. Slide chemical treatment, array production, LDR protocol, and hybridization conditions were performed as previously reported (Candela et al., 2012). Briefly, LDR reactions were carried out in a final volume of 20 μl containing 500 fmol of each LDR-UA HTF-Microbi.Array probe, 50 fmol of PCR product, and 25 fmol of the synthetic template (5′-AGC CGCGAACACCACGATCGACCGGCGCGCGCAGCTGCAGCTTGC TCATG-3′). LDR products were hybridized on universal arrays, setting the probe annealing temperature at 60°C. All arrays were scanned and processed according to the protocol and parameters already described. Fluorescence intensities were normalized on the basis of the synthetic ligation control signal. The relative abundance of each bacterial group was obtained by calculating the relative fluorescence contribution of the corresponding HTF-Microbi.Array probe as a percentage of the total fluorescence.

### RNA extraction and real-time PCR

Colon specimens were collected immediately after sacrifice and total RNA was extracted using Trizol® reagent (Life Technologies, CA, USA) according to the manufacturer's instructions. Extracted RNA samples were treated with DNase I to remove any genomic DNA contamination using DNA-free kit (Ambion, USA) and reverse-transcripted using RevertAid™ First Strand cDNA Synthesis Kit (Fermentas, Canada). COX-2 and β-actin mRNAs were reverse-transcribed using random hexamer primers (Fermentas, Canada). COX-2 and β-actin mRNA levels were analyzed by real-time PCR using SYBR® Select Master Mix (Life Technologies, CA, USA) and StepOnePlusTM system (Applied Biosystems, CA, USA) according to the manufacturers' instructions. The melting curve data were collected to check PCR specificity. Each cDNA sample was analyzed in triplicate. COX-2 mRNA levels were normalized against β-actin mRNA and relative expressions were calculated using the 2-2ΔCt formula. COX-2 primer pair: 5′- TTC TCT ACA ACA ACT CCA TCC TC -3′ and 5′- GCA GCC ATT TCC TTC TCT CC -3′ (247 bp product); β-actin primer pair: 5′- ACC AAC TGG GAC GAC ATG GAG -3′ and 5′- GTG GTG GTG AAG CTG TAG CC -3′ (380 bp product).

### Data analysis

Statistical analysis was carried out using GraphPad Prism 6 (GraphPad Software Inc., San Diego, CA, USA). Data are expressed as mean ± SEM of at least three independent determinations. Student's *t*-test, analysis of variance (one-way ANOVA) followed by Bonferroni's *post-hoc* test for multiple comparison were used to assess the statistical significance of the differences. Differences were considered statistically significant at *P* < 0.05. Euclidean distance of HTF-Microbi.Array relative abundance profiles were used to perform PCoA and analysis was accomplished using the R packages Made4, Vegan, and Stats (www.cran.org).

## Results

### Clinical colitis activity

**Figure 2:**
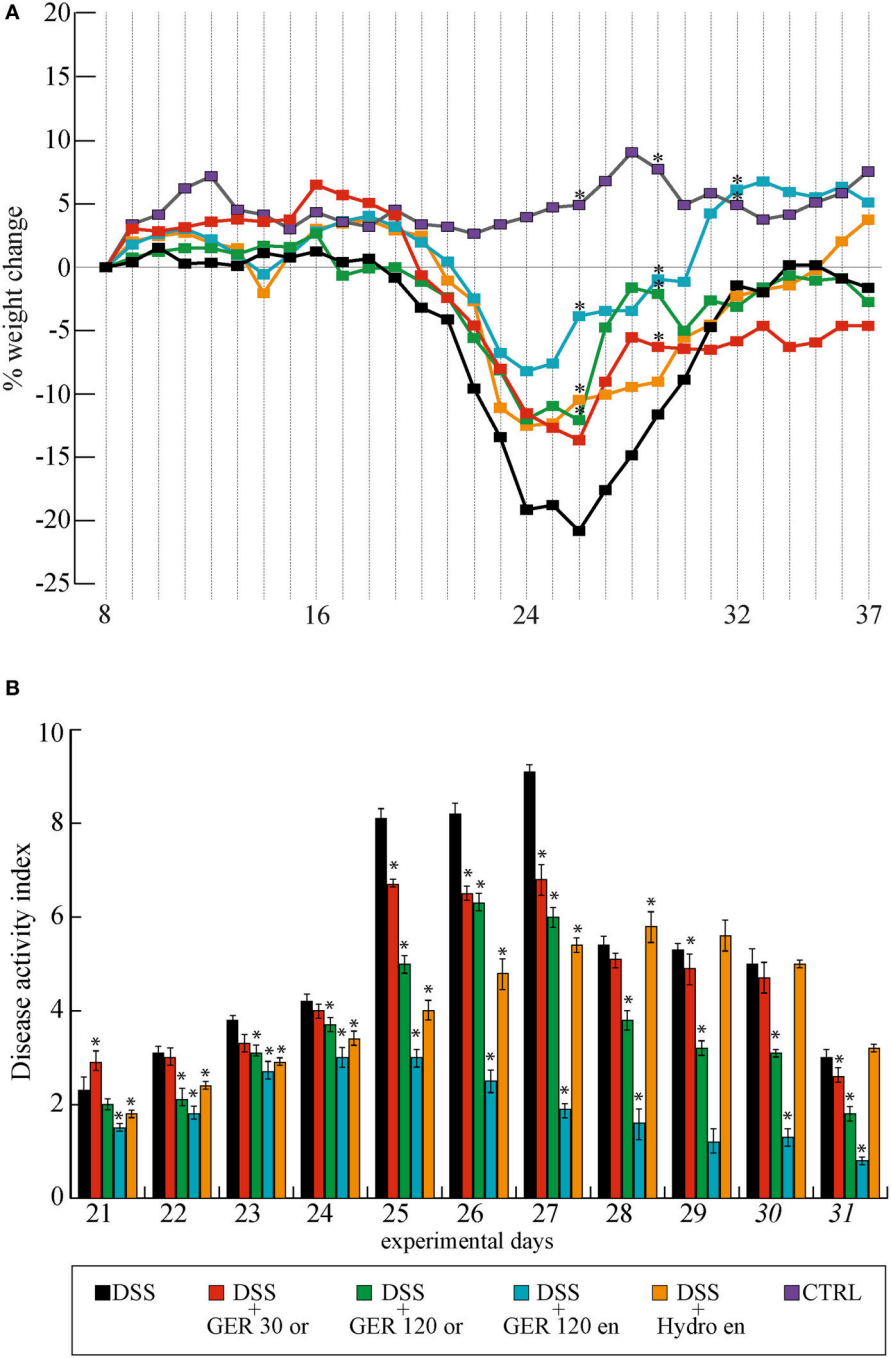
**Weight change percentage (A) and disease activity index (DAI) score of colitis (B) in different mice experimental groups**. Maximum DAI score was reached between days 25 and 27. Maximum weight loss (22%) was recorded between days 22 and 27. Weight recovery ends at days 37. Data are expressed as mean ± SD. Analysis of variance (one way-ANOVA) was performed (for weight changes only at days 26, 29, and 32) to assess the statistical significance of the differences. ^*^P < 0.05 if compared to DSS group mean values. Statistical significance for DAI score differences (analysis of variance, one way-ANOVA) are reported in Supplementary Table 1.

The effect of DSS and DSS-Ge-OH treatments was evaluated considering the DAI calculated as the sum of weight loss, stool consistency, and blending scores (Table 1). All DSS-treated mice started to show mild clinical signs of disease 2 days before the end of the 1.5% DSS treatment (day 21) due to the simultaneous increase in stool consistency index and bleeding index (maximum DAI score = 2.3). The most evident clinical signs of each group were recorded between days 25 and 27 (Figure 2) with a maximum DAI score of 9.1 for the DSS group and with severe weight loss that peaked between days 25 and 28 (Figure 2A). Ge-OH at 30 mg kg^(−1)^ reduced the DAI score of colitis during the acute phase but did not affect this index during the recovery phase (Figure 2B). At this Ge-OH dose, the DAI score maintained the same trend observed in DSS-treated mice. At the higher oral dose, Ge-OH reduced the DAI score for almost the entire duration of colitis and especially during the recovery phase. Statistical analysis of data in Figure 2B are provided in Supplementary Table 1. These positive Ge-OH effects were further enhanced when colitic mice were treated with enema-administered Ge-OH, resulting in a very low weight loss and a strongly reduced DAI score for the whole duration of colitis.

### Inflammatory cytokine profile of colitis

**Figure 3:**
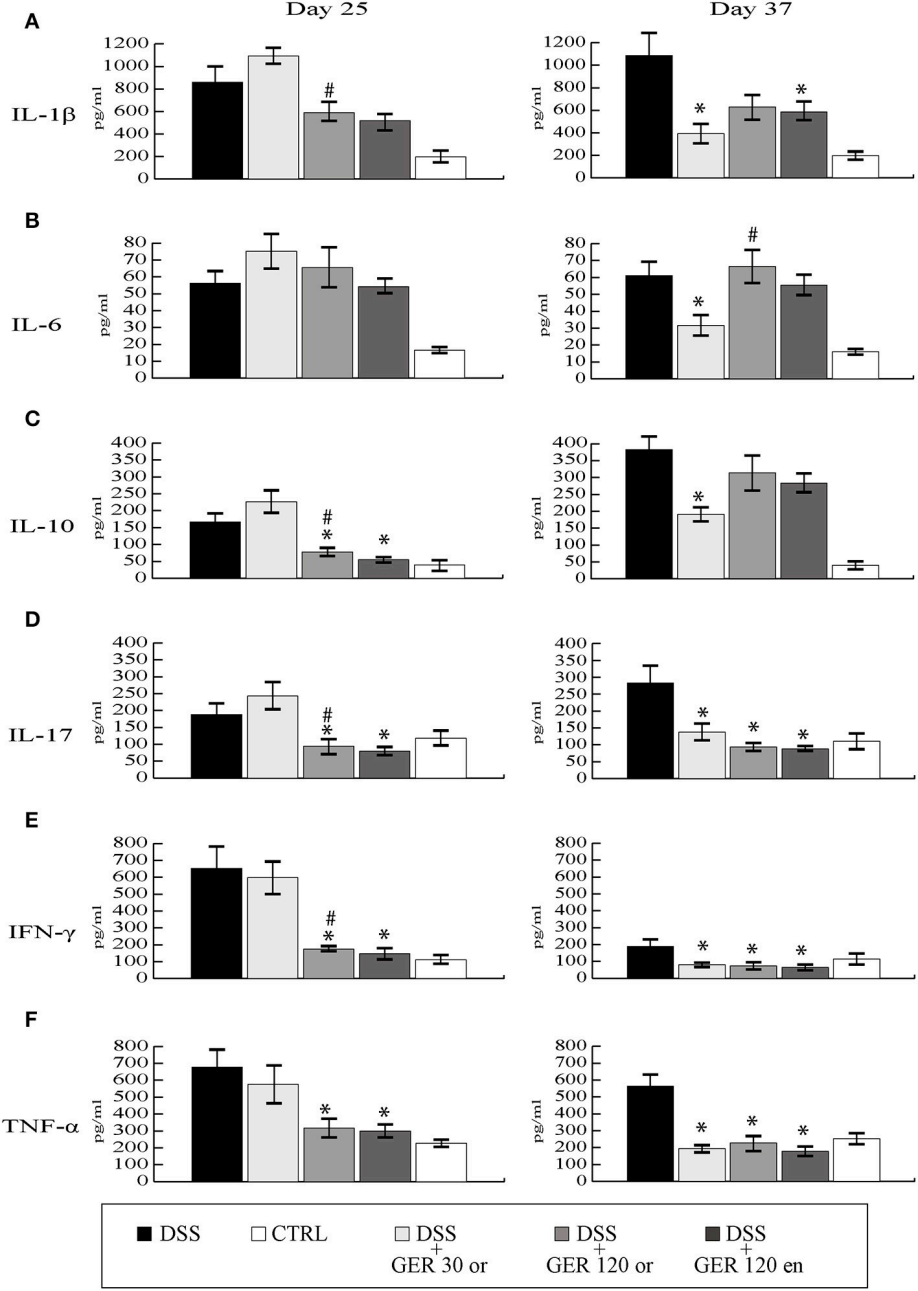
**Plasma cytokine variations during experimental colitis, measured at days 25 and 37**. Cytokines were determined using a 6-plex mouse bead immunoassay kit. Levels of IL-1β **(A)**, IL-6 **(B)**, IL-10 **(C)**, IL-17A **(D)**, IFN-γ **(E)**, and TNFα **(F)** are shown. Data are expressed as mean ± SEM of at least three replicates (*n* = 9). ^#^*P* < 0.05 in the comparison between to Ge-OH 30 and Ge-OH 120 or groups. ^*^P < 0.05 if compared to DSS group.

Plasma levels of IL-1β, IL-6, IL-10, IL-17, TNFα, and IFNγ were detected in blood samples from all experimental mice group at two different time points, one corresponding to the acute phase of colitis (day 25), and one at the end of the recovery phase (day 37). DSS treatment significantly increased (*P* < 0.05) all the cytokines measured, both at day 25 and day 37 (Figure 3). At day 25, oral administration of Ge-OH at the lower dose of 30 mg/mg kg^(−1)^ did not modify the inflammatory profile of DSS-treated mice. Oral administration of the higher Ge-OH dose of 120 mg kg^(−1)^ and Ge-OH 120 mg kg^(−1)^ enema administration significantly decreased IL-10, IL-17, TNFα, and IFNγ (*P* < 0.05), but neither IL-1β nor IL-6. At day 37 when colitis tended to become chronic, Ge-OH-treated mice showed a better inflammatory profile than DSS-treated mice. In particular, the lower dose of oral Ge-OH significantly reduced all the measured cytokines (*P* < 0.05). The higher oral dose and enema administration of Ge-OH significantly decreased IL-1β, IL-17, IFNγ, and TNFα (*P* < 0.05) but neither IL-6 nor IL-10.

### Histological evaluation of colitis

**Figure 4:**
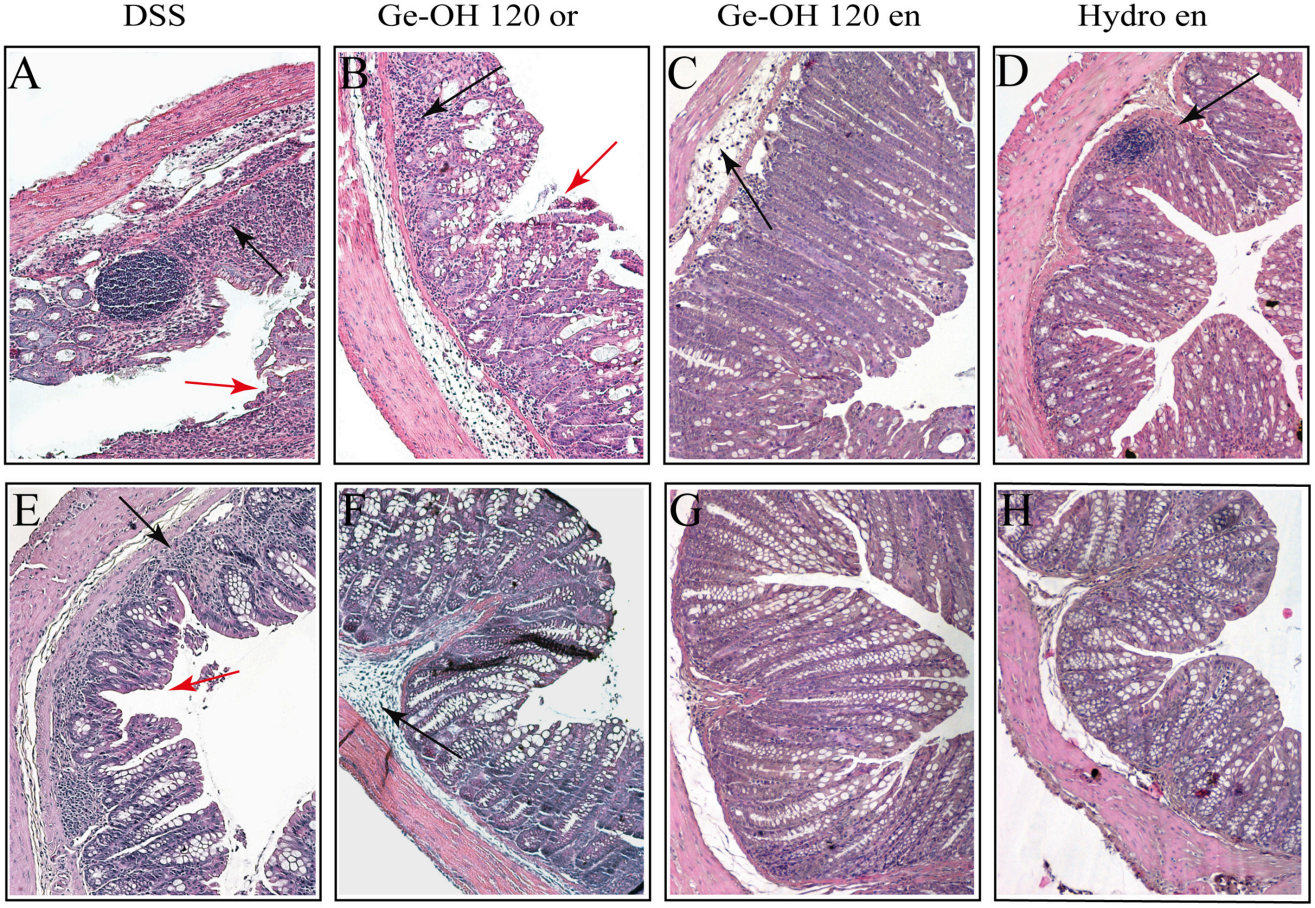
**Differences in histological architecture induced by Ge-OH 120mg kg^**(−1)**^ and hydrocortisone 2.5 mg kg^**(−1)**^ during the experimental colitis**. Colon specimens were collected from mice on days 25 **(A–D)** and 37 **(E–H)**. Histopathological changes in individual crypts are shown in representative hematoxylin and eosin-stained sections. Red arrows indicate loss of crypt architecture associated with epithelial damage and flattened villi while black arrows indicate leukocyte infiltration (Magnification: 10X; bar = 100 μm).

Histological evaluation of the colon was made from the colocecal junction to the anus. Overall, the tissue damage tended to be limited to the terminal colon and rectum regions, and can be classified as moderate colitis (Figure 4). At day 25 (Figures 4A–C), the colon mucosa in the DSS-treated mice showed a diffuse loss of goblet cells, focal crypt abscesses, diffuse hyperemia, moderate cellular infiltration in the mucosa, and focal epithelial erosions. Diffuse hyperemia, mild loss of goblet cells, mild cellular infiltration but no crypt abscesses, or epithelial erosions were also present in the mucosa of oral Ge-OH-treated mice at both doses administered [see Supplementary Figure 1 for Ge-OH 30mg kg^(−1)^]. The colon in the Ge-OH enema-treated mice was characterized by a lower mucosa distortion (elongation) and showed moderate loss of epithelium, and low leukocyte infiltrations.

After weight recovery (day 37), the colon mucosa in the DSS-treated mice showed a diffuse loss of goblet cells, focal crypt abscesses, diffuse hyperemia, and mild cellular infiltration (Figure 4D), while the mucosa of oral Ge-OH-treated mice presented diffuse hyperemia but a milder loss of goblet cells, a milder cellular infiltration, and no crypt abscesses at with dose administered (Figures 4E,F). Colon mucosa in the enema Ge-OH-treated mice showed a normal architecture similar to that of healthy controls. In conclusion, histological and clinical improvements were evident in the Ge-OH-treated mice and particularly in the enema-treated animals.

### Ge-OH-induced microbiota modifications

**Figure 5:**
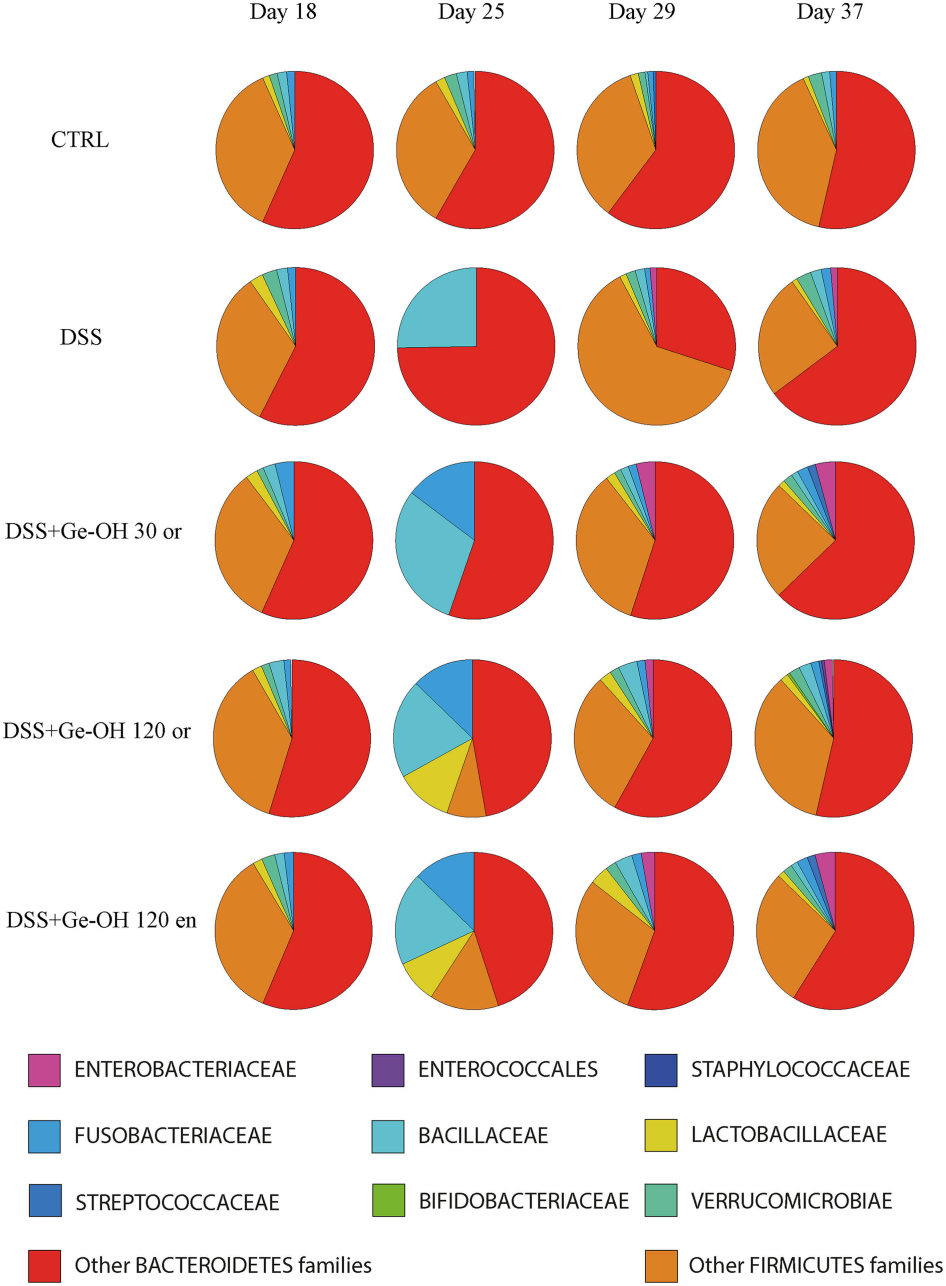
**Temporal dynamics at the family level of the fecal microbial community of dextran sulfate sodium (DSS)-treated mice**. The microbiota composition of healthy mice (CTRL), colitic mice (DSS), colitic geraniol orally treated mice [Ge-OH 30 mg kg^(−1)^, 120 mg kg^(−1)^], and colitic geraniol enema-treated mice [120 mg kg^(−1)^] is shown. Other Bacteriodetes and Firmicutes families that are not are listed separately have been combined into a single group. The microbiota composition of the mice group treated with Ge-OH-free oral suspension or Ge-OH-free enema suspension showed no differences from those of the healthy mice group.

Since the Ge-OH-free suspension itself did not induce microbiota alterations, we investigated the impact of Ge-OH treatment on DSS-induced microbiota dysbiosis in mice. Mice stools were collected on days 18, 25, 29, and 37. Figure 5 shows the phylogenetic structure of the intestinal microbiota characterized using the HTF-Microbi.Array universal platform. DSS treatment prompted profound, progressive, and transient changes in mice microbiota composition, compared to colitis-negative controls (group I), defining a peculiar microbiota trajectory during the induced colitis. In particular, on day 18, after 1 day of DSS treatment, the overall microbiota structure of DSS mice still resembled that of control mice. At day 25, after seven days of DSS, we observed a global temporary restructuring of the intestinal microbiota composition. At day 29 a transitory reduction of *Bacteroidetes* associated with an increase in Firmicutes was recorded. However, on day 37, DSS-treated mice recovered a microbiota structure similar to that of healthy controls.

While oral Ge-OH treatment at 30 mg kg^(−1)^ exerted only a mild impact on the temporal dynamics of DSS-induced microbiota dysbiosis, oral and enema treatment at a dose of 120 mg kg^(−1)^ resulted in considerable protection against the transient DSS-dependent reduction of *Bacteroidetes*, favoring a faster recovery of a community profile similar to that of healthy controls. In particular, on day 25, Ge-OH at 120 mg kg^(−1)^ (both enema and orally administered) triggered a Lactobacillaceae increase that reached a relative abundance of 11.2 and 9.7% respectively, notably higher than the corresponding value in control mice. This Ge-OH-dependent high relative abundance of Lactobacillaceae was maintained until day 29 after which Ge-OH-treated mice permanently recovered from the DSS-induced reduction of *Bacteroidetes* 8 days earlier with respect to the corresponding DSS-treated mice. These effects are certainly related to the antibacterial action of Ge-OH, evidenced by its low minimal inhibitory concentration (MIC) on model bacteria species (see Supplementary Table 2). Differently from what observed in DSS treated mice, in healthy mice Ge-OH treatment, even at the dose of 120 mg kg^(−1)^ (orally administered), did not produced the same marked changes in the microbiota. Indeed, the microbiota composition of mice treated with Ge-OH 120 mg kg^(−1)^ showed a slight increase in Lactobacillaceae, Bacillaceae and Bacteroidetes families (see Supplementary Figure 2).

### Down-regulation of COX-2 through Ge-OH treatment

**Figure 6:**
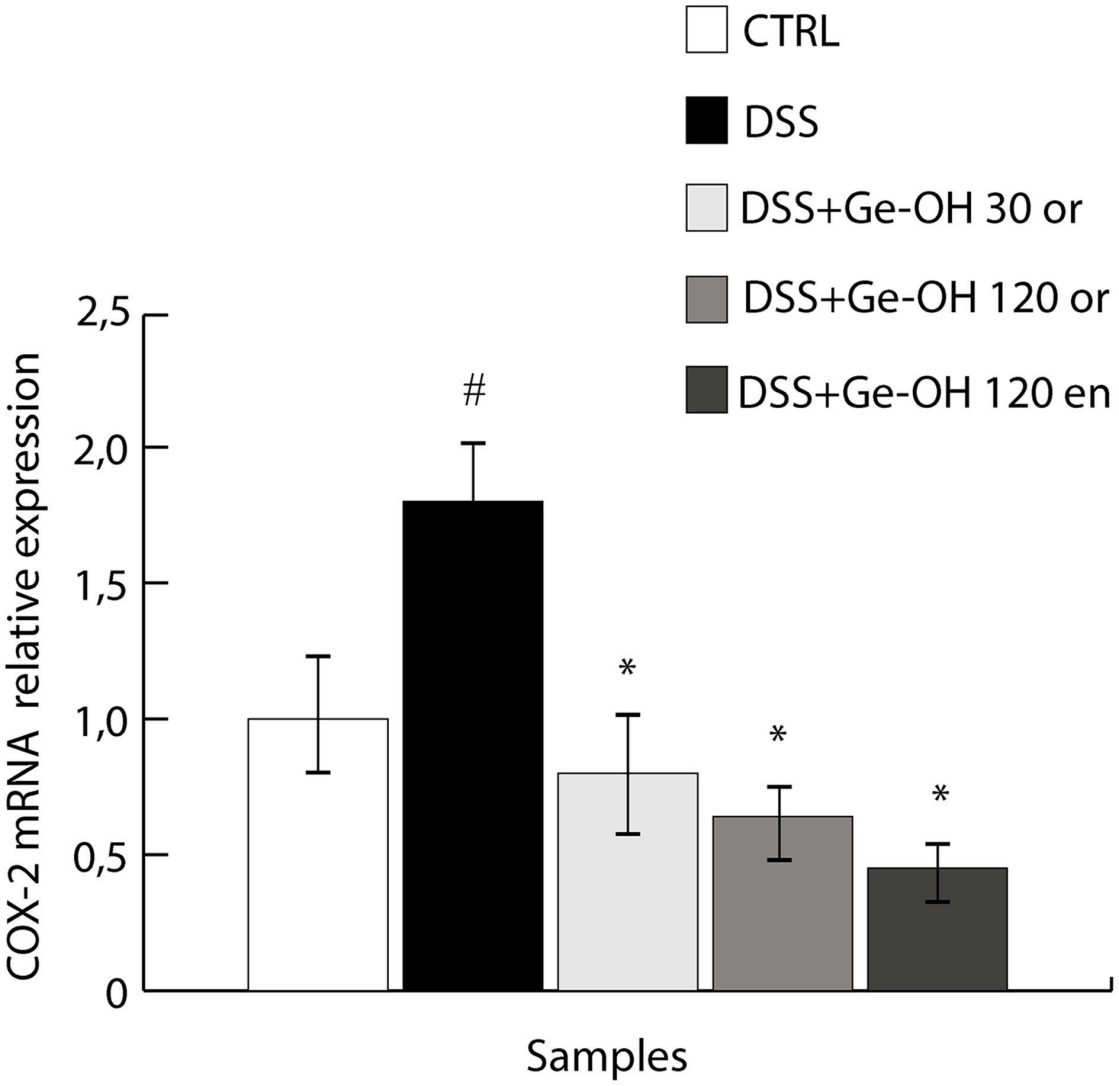
**Geraniol (Ge-OH) modulates cyclooxygenase-2 (COX-2) expression ***in vivo*** in colon specimens during the acute phase (day 25)**. COX-2 mRNA expression was evaluated by real-time PCR. COX-2 mRNA levels were normalized against β-actin mRNA and relative expressions were calculated using the 2-2ΔCt formula. COX-2 overexpression induced by DSS was significantly reduced by Ge-OH treatment. Data are expressed as mean ± SEM of at least three replicates (*n* = 6). ^*^P < 0.05 compared to DSS group. ^#^*P* < 0.05 compared to CTRL group.

Since COX-2 plays a crucial role in the production of many lipid mediators involved in intestinal inflammation and is one of the major targets of IBD pharmacological therapy, we analyzed COX-2 mRNA expression in colon tissues during DSS-induced colitis (Figure 6). Our data support the previously reported finding that COX-2 mRNA significantly increases in the gut wall of DSS-treated mice (De Fazio et al., 2014). At day 25, we observed a significant increase (1.8-fold, *P* < 0.05) in COX-2 expression in the gut wall of DSS-treated mice. Ge-OH decrease the COX-2 expression in DSS treated mice returning it to values comparable to those of the control.

## Discussion

Inflammatory bowel disease (IBD) comprises a group of chronic inflammatory conditions affecting the gastrointestinal tract. The mucosal immune system of IBD patients has lost the ability to self-regulate and remains chronically activated. IBD is a well-established risk factor for colon cancer (CRC) development, with an increasing incidence linked to younger age at IBD diagnosis, longer IBD duration, and more severe intestinal inflammation. Conventional IBD therapies include COX-2 inhibitors (aminosalicylates and their derivatives), corticosteroids, immunomodulatory drugs, antibiotics, and biologic drugs such as the monoclonal antibody against tumor necrosis factor alpha (TNFα), a pivotal pro-inflammatory cytokine able to start and maintain the inflammatory process in the gut. Besides antibiotics, probiotics have also been used in the treatment of ulcerative colitis to counteract dysbiosis (Bibiloni et al., 2005). Since, IBD usually relapses, all these therapies require long-term administration.

Ge-OH is a non-toxic compound, classified as Generally Recognized As Safe (GRAS) by the US Food and Drug Administration. The European Food Security Agency (EFSA) hazard assessment conclusion for Ge-OH established a Derived No Effect Level (DNEL) of 13.5 mg kg^(−1)^ for humans (General Population—Hazard via oral route), corresponding to 100–120 mg kg^(−1)^ in mice. Ge-OH is currently receiving substantial attention for its antitumorigenic, anti-inflammatory, and antimicrobial effects that have been clearly demonstrated *in vitro*. Nevertheless, its role as an anti-dysbiotic agent in colon inflammation has never been investigated. Our study adopted a mouse model of DSS-induced moderate to severe colitis to evaluate the antimicrobial and anti-inflammatory therapeutic activity of Ge-OH doses considered safe.

Ge-OH, orally administered at 30 and 120 mg kg^(−1)^ halved the mice weight loss and reduced the disease activity index (DAI) of colitis. At histological level, Ge-OH was able to preserve crypt architecture and decrease leukocyte infiltration, with a much more evident effect at the higher dose (both enema or orally administered). Moreover, enema-administered Ge-OH strongly improved signs of colitis maintaining a lower DAI and preserving colon mucosa integrity. These clinical observations are further supported by a significant reduction of COX-2 mRNA expression in the colonic mucosa of Ge-OH-treated mice.

Circulating cytokine levels are indicative of the overall inflammatory status of animals, with IL-1, IL-6, IL-17, and TNFα playing a key role in the pathogenesis of IBD (Muzes et al., 2012). TNFα is a master cytokine in IBD pathogenesis and its orchestrating role in colonic inflammation is confirmed by the efficacy of anti-TNFα therapy in IBD patients (Chaparro et al., 2012). The circulating TNFα level correlates with clinical activity both in ulcerative colitis and Crohn's disease (Bibiloni et al., 2005) and increases in acute phases of DSS colitis (Alex et al., 2009). So, while circulating TNFα and IL-17 levels seem to correlate with the DSS colitis clinical course, IL-1β, and IL-10 mainly correlate with the histological damage that tends to become chronic (Alex et al., 2009; De Fazio et al., 2014). The higher oral dose of Ge-OH significantly reduced circulating TNFα and IL-17 in Ge-OH-treated mice after weight recovery at the end of the experiments. This decrease was equally evident after Ge-OH enema administration. These results are in agreement with those obtained by Medicherla et al. (2015) who found a significantly reduced expression of the major pro-inflammatory cytokines in the colon specimens (TNF-α, IL-1β, and IL-6), associated with reduced total and nuclear amounts of NF-κB (p65) after oral administration of Ge-OH [50 and 100 mg Kg ^(−1)^]. They also identified an antioxidant activity of Ge-OH at colon level, evaluated as a decrease in lipid peroxidation marker.

DSS treatment compromises gut microbiota homeostasis, resulting in a dysbiosis characterized by a transient reduction of dominant mutualistic microbiota components such as *Bacteroidetes*, confirming previous findings (Nagalingam et al., 2011). Ge-OH oral and enema treatment at 120 mg kg^(−1)^ protects DSS-treated mice against this transient reduction of *Bacteroidetes*, boosting a faster recovery of a healthy microbiota profile. Interestingly, 120 mg kg^(−1)^ geraniol-treated mice presented a transient increase in the relative abundance of *Lactobacillaceae* from day 25 to day 29. This raises the question of whether the transient Ge-OH-dependent increase in *Lactobacillaceae*, heralding the recovery of a healthy profile, is somehow involved in promoting a faster recovery from DSS-associated dysbiosis. Dysbiosis always includes a decreased bacterial biodiversity (Honda and Littman, 2012). GeOH 120 mg kg^(−1)^ treatment was able to increase bacterial biodiversity in DSS-treated mice starting from the 10th day of Ge-OH assumption. It is likely that the decreased inflammation we observed in Ge-OH oral and enema-treated mice during colitis recovery is also due to the healthy microbiota status found in these mice.

The central finding of this study in a colitis model is the multi-target effect of Ge-OH treatment that simultaneously targeted dysbiosis, local, and systemic inflammation and mucosal damage. The decreased activity of COX-2 in colon specimens is a clear demonstration of the anti-inflammatory effect of Ge-OH that contributes to the decreased mucosal damage. This effect certainly involves the colonic mucosa, even if it is reasonable to assume that *in vivo* Ge-OH may also target COX-2 expression in immune system cells inside the colon wall (Su et al., 2010).

## Conclusions

IBD therapy is based on the use of anti-inflammatory molecules and immunomodulatory agents that act by strongly and non-specifically inhibiting the inflammatory response but their long-term use might trigger the onset of severe side-effects. The effects of Ge-OH could be of great importance in the treatment of human IBD. Since geraniol's antimicrobial effect does not seem to induce bacterial resistance, a phenomenon commonly observed with conventional antibiotic drugs, it would be very interesting to ascertain whether Ge-OH is able to control dysbiosis and inflammatory status in human IBD patients. In addition, Ge-OH's anti-tumor activities could help reduce the risk of CRC in IBD patients. Thus, this investigation represents a preclinical assessment prior to developing further studies on the effects of oral and enema Ge-OH administration in patients with gut inflammation and/or dysbiosis. Since Ge-OH has its peak therapeutic effect on colitis when directly administered into the colon, it is of great importance to find oral delivery systems able to inhibit intestinal Ge-OH absorption after its oral administration. On the contrary, Ge-OH without any delivery system may be orally administered to obtain a systemic anti-inflammatory effect or to target other organs, such as brain.

## Author contributions

Participated in research design: LD, MV, MC, FR, and ES. Conducted the experiments: LD, MV, AS, EC, and CR. Performed data analysis: MC, ES, and MC. Wrote the manuscript: LD and ES.

### Conflict of interest statement

The authors declare that the research was conducted in the absence of any commercial or financial relationships that could be construed as a potential conflict of interest.

## Abbreviations

Ge-OH: geraniol
DSS: Dextran sulfate sodium
IBD: Inflammatory bowel disease
COX-2: Cyclooxygenase-2
CRC: Colorectal cancer.
