# Supplementary material for: Dietary Geraniol by Oral or Enema Administration Strongly Reduces Dysbiosis and Systemic Inflammation in Dextran Sulfate Sodium-Treated Mice
Source: Front Pharmacol. 2016 Mar 3;7:38. doi: 10.3389/fphar.2016.00038 (PMC4776160; doi:10.3389/fphar.2016.00038)
Supplement: Supplementary file 1 [file DataSheet1.doc]

Supplementary Material:

**Dietary geraniol orally- or enema-administered strongly reduces dysbiosis and systemic inflammation in dextran sulphate sodium-treated mice.**

De Fazio L^1^, Valerii MC^1^, Cavazza E^1^, Strillacci A^1^, Candela M^2^, Centanni M^2^, Ricci C^3^, Rizzello F^4^, Campieri M^4^ and Spisni E^1*^

Corresponding author: Enzo Spisni

[enzo.spisni@unibo.it](mailto:enzo.spisni@unibo.it)

**SUPPLEMENTARY TABLE AND FIGURES**

**Supplementary** **Table 1**


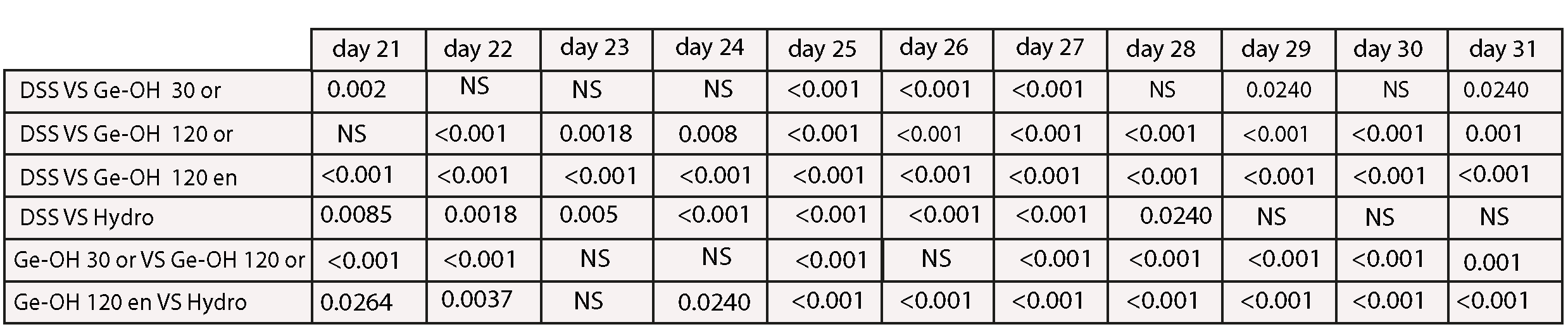


**Supplementary** **Table 1.** Statistical analysis of Disease Activity Index (DAI): differences between groups were determined by one-way analysis of variance followed by Bonferroni’s post-hoc test for multiple comparison. All analyses were performed using GraphPad Prism 6 software (GraphPad Software Inc., San Diego, CA, USA). p <0.05 and p<0.001 were considered to indicate statistical significance. NS, not significant.

**Supplementary** **Table 2**


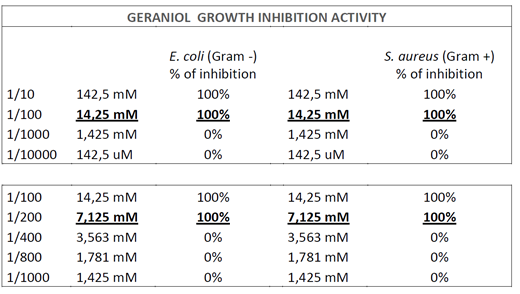


**Supplementary** **Table 2.** The minimal inhibitory concentration (MIC) of Ge-OH was evaluated using the species E. coli and S. aureus as model species of Gram-negative and Gram-positive microorganisms, respectively. MIC is expressed in bold.

The minimal inhibitory concentration (MIC) was evaluated on 5 ml Luria Bertani (LB) agar plates using the species *E. coli* and *S. aureus* as model species of Gram-negative and Gram-positive microorganisms, respectively. 10^6^ CFU of exponential phase microbial cells were plated on LB plates containing geometric dilutions of each compound and incubated for 12 h at 37°C in aerobic conditions. For each compound the dilutions tested ranged between 200 mM and 100 μM and the MIC was considered the minimal concentration inhibiting 100% microbial growth.

**Supplementary** **Figure 1**

**
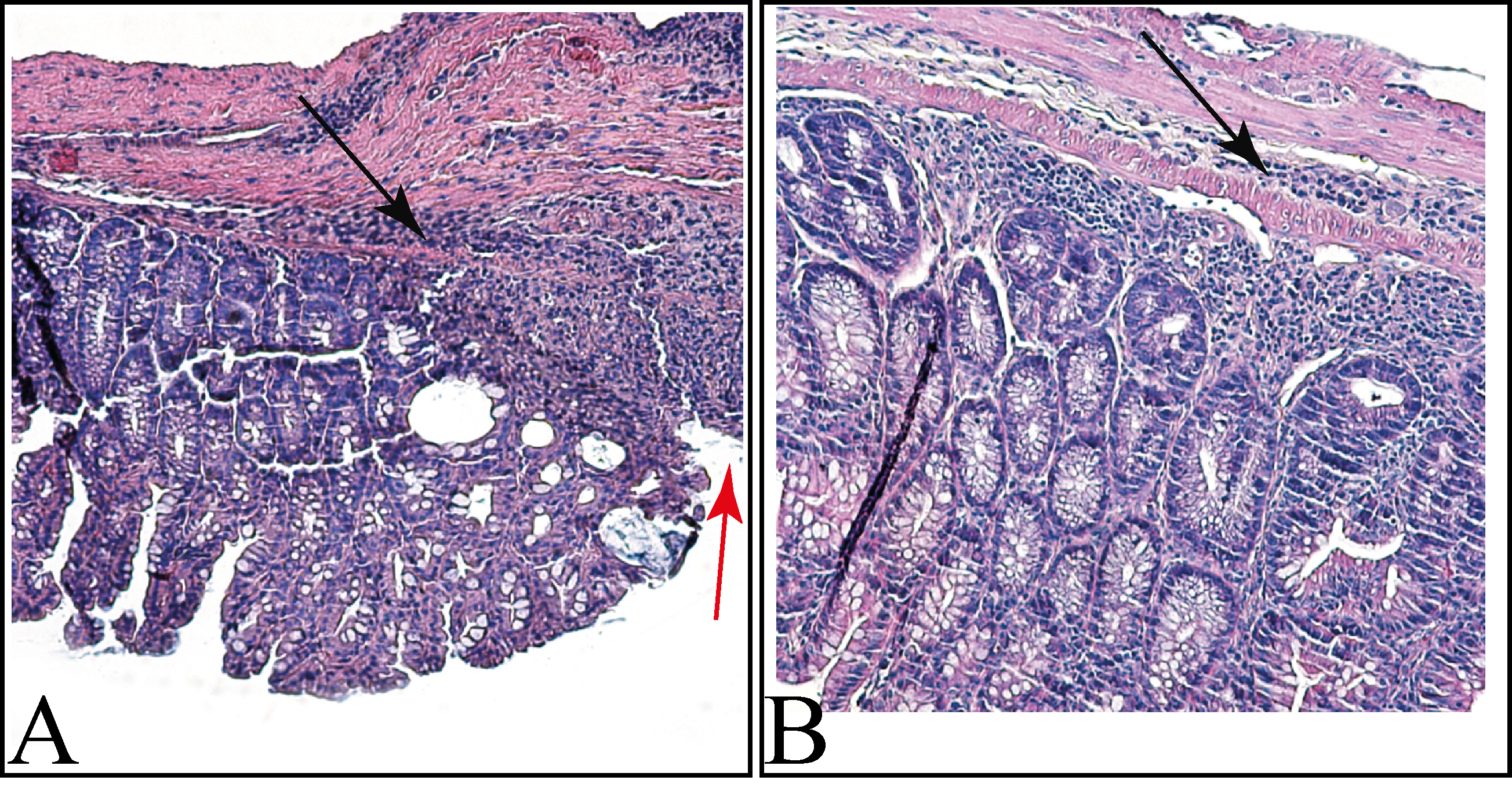
**

**Supplementary** **Figure 1.** Differences in histological architecture induced by Ge-OH 30mg kg^(-1)^ during the experimental colitis. Colon specimens were collected from mice on days 25 (A) and 37 (B). Histopathological changes in individual crypts are shown in representative hematoxylin and eosin-stained sections. Red arrows indicate loss of crypt architecture associated with epithelial damage and flattened villi while black arrows indicate leukocyte infiltration (Magnification: 10X).

**Supplementary** **Figure 2**

**
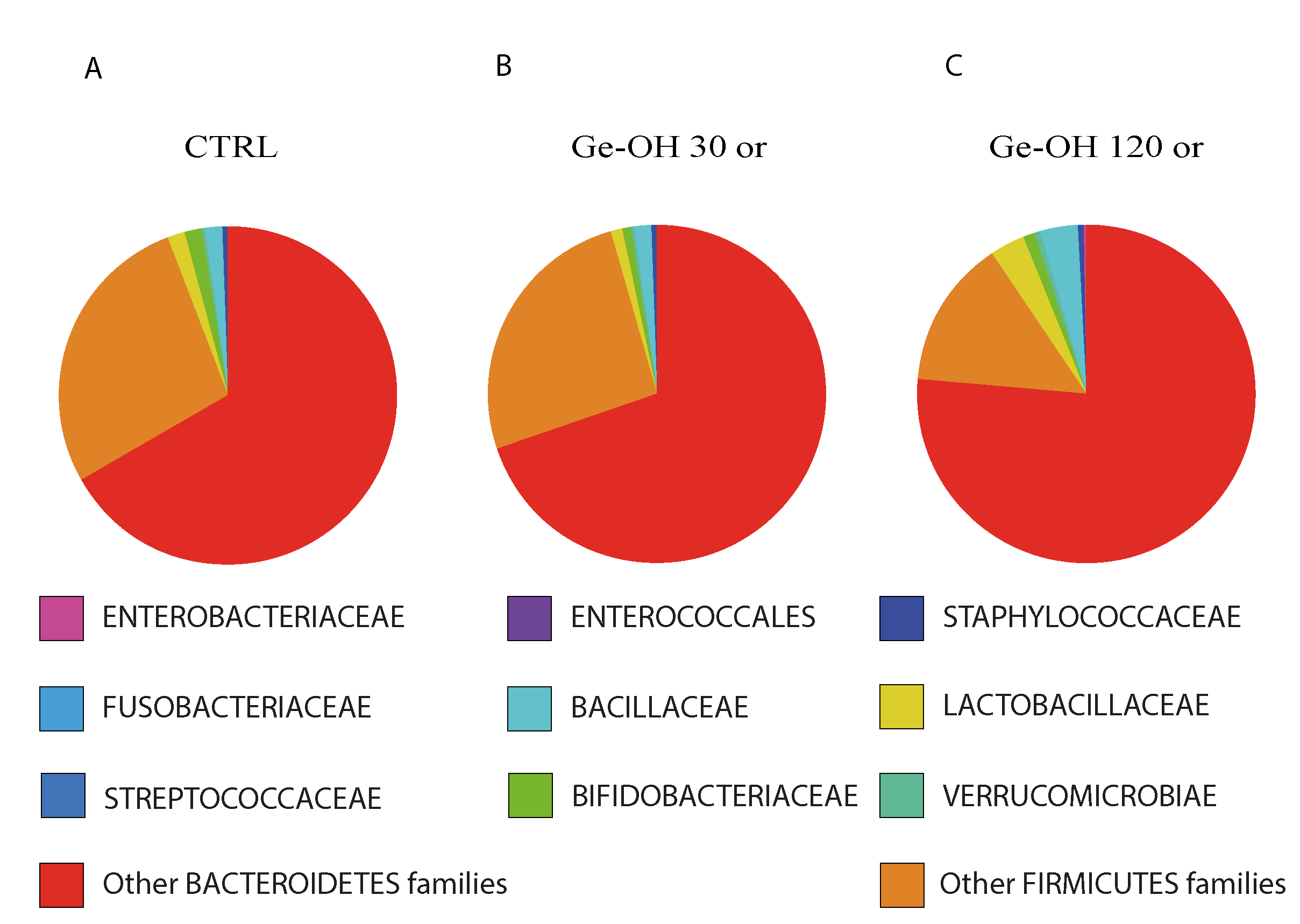
**

**Supplementary** **Figure 2**. Temporal dynamics of the fecal microbial community, at the family level, of healthy mice treated with orally administered Ge-OH, characterized using the HTF-Microbi.Array universal platform. Mice stools were collected after 17 days of oral administration of Ge-OH-free suspension (panel A), Ge-OH 30 mg kg^(-1)^ (panel B) and Ge-OH 120 mg kg^(-1)^ (panel C). Other Bacteriodetes and Firmicutes families that are not are listed separately have been combined into a single group. The microbiota composition of the group treated with Ge-OH 30 mg kg^(-1)^  showed no difference from that of control mice treated with Ge-OH-free oral suspension. The microbiota composition of mice treated with Ge-OH 120 mg kg^(-1)^ showed an increase in *Bacteroidetes.* It also showed a Lactobacillaceae and Bacillaceae increase, even if the other Firmicutes families were reduced.
